# Promegestone Prevents Lipopolysaccharide-Induced Cervical Remodeling in Pregnant Mice

**DOI:** 10.3390/cells14040242

**Published:** 2025-02-07

**Authors:** Amna Nadeem, Lubna Nadeem, Stephen James Lye, Oksana Shynlova

**Affiliations:** 1 Department of Physiology, University of Toronto, Toronto, ON M5S 1A8, Canada; anadeem1@sgu.edu (A.N.); lye@lunenfeld.ca (S.J.L.); 2 Sinai Health System, Lunenfeld-Tanenbaum Research Institute, Toronto, ON M5G 1X5, Canada; nadeem@lunenfeld.ca; 3 Department of Obstetrics and Gynecology, University of Toronto, Toronto, ON M5G 1E2, Canada

**Keywords:** uterus, preterm labor, progesterone, cervical remodeling, promegestone/R5020, 20α-HSD, cytokines, mouse models, lipopolysaccharide (LPS), steroid metabolism

## Abstract

In most mammals, a withdrawal of the pro-gestational hormone progesterone (P4) is necessary for labor onset. In murine cervix, P4 withdrawal is mediated by enzymes steroid 5-alpha-reductase type 1 (SRD5A1) and 20-alpha-hydroxysteroid-dehydrogenase (20α-HSD). Previously, we have shown that inflammatory stimuli induce 20α-HSD levels in uterine muscle (myometrium). Here, we hypothesized that (1) infectious inflammation alters the levels of both P4-metabolizing enzymes in mouse cervix, which consequently ceases P4-mediated inhibition of cervical remodeling, thereby inducing preterm labor (PTL); (2) a progestin, selective progesterone receptor modulator promegestone (aka R5020), non-metabolizable by 20α-HSD, can block lipopolysaccharide (LPS)-induced PTL in mice by maintaining P4 signaling and preventing cervical remodeling. Using RT-PCR and IHC/IF methods, we evaluated the effect of inflammation on the expression of both enzymes in mouse cervix and determined if R5020 can prevent cervical remodeling and PTL in mice. We found significant induction of SRD5A1 and 20α-HSD proteins (*p* < 0.01), as well as transcript levels of pro-inflammatory cytokines *Il1b*, *Il6*, chemokines *Cxcl1*, *Ccl2,* cervical ripening enzyme *Has2*, hyaluronic acid binding protein/HABP (*p* < 0.05), and a simultaneous decrease in major extracellular fibrillar proteins, collagen type 1 and type 3 (*col1a1, col3a1*), in mouse cervix during PTL. The prophylactic administration of R5020 in pregnant mice significantly inhibited cervical remodeling and prevented PTL irrespective of the route of LPS-induction, systemic or local. We concluded that R5020 is a promising novel drug application for preterm birth prevention.

## 1. Introduction

Human gestation is a unique process ensuring safe fetal development inside the womb until full term. Successful parturition at term depends on two complementary processes: (1) the transformation of a uterine smooth muscle (myometrium), which is quiescent throughout gestation, to a strong contractile unit capable of forceful contractions to expel the mature fetus during term labor (TL) [1], and (2) the transition of a rigid, closed uterine neck (cervix) to a soft dilated conduit, allowing the safe passage of the baby into the extra-uterine environment [2]. Until recently, it was assumed that the cervix is a passive organ with homogenous microstructure [3]. Our own research employing MRI and studies of others using optical coherence tomography shows the complex architecture of the cervix, with the internal region adjacent to uterine body (endocervix) being different from the external region (ectocervix) [4,5,6,7]. Moreover, we see evidence that the uterine endocervix has a distinct sphincter-like structure that maintains gate-keeping function during pregnancy [4].

Progesterone (P4) is the key pro-gestational steroid hormone which maintains the quiescence of uterine muscle and suppresses cervical remodeling throughout pregnancy [8,9]. P4 affects cervical, decidual, and myometrial cells, and its action is mediated through the progesterone receptors (PRs) [10,11]. P4 withdrawal triggers the onset of term and preterm labor [8]. In lower mammals P4 withdrawal is achieved by luteolysis, leading to a fall in maternal systemic hormone levels [12]. In women, circulating P4 levels remain elevated throughout gestation, declining only after delivery of the placenta [13]. In the myometrium, the intracellular P4 withdrawal occurs due to an increased level and activity of the P4-metabolising enzyme aldo-keto reductase family 1 member C1 (encoded by the *AKR1C1* gene in humans, *Akr1c18* in mice, translating to 20α-HSD protein) [14,15], which converts active P4 into inactive metabolite 20-alpha-hydroxyprogesterone/20αOHP. In mouse myometrium, 20α-HSD expression and activity are increased during TL and PTL [14], suggesting a local regulation of P4 metabolism.

The process of cervical remodeling is similar between humans and rodents [16]. In mice, cervical ripening is initiated at late gestation, 2–4 days before TL (i.e., on gestational day (GD) 15–16), when systemic P4 levels are high [16]. In the murine cervix, the dominant enzyme for steroid metabolism is 5α-reductase type 1 (SRD5A1) expressed in luminal epithelium and stromal fibroblasts, which shows functional homology to mouse 20α-HSD [17]. SRD5A1 converts active P4 into inactive metabolite 5α-dihydro-progesterone/5αDHP, thereby reducing its availability and attenuating P4 signaling [17]. Both P4-metabolozing enzymes (20α-HSD and SRD5A1) were detected in mouse cervix; importantly, cervical SRD5A1 expression is upregulated near term [17]. A knockout of SRD5A1 in mice causes parturition defects due to impaired cervical ripening and accumulation of P4 in the cervical tissue [17]. It has been shown that the P4 receptor antagonist RU486 induces cervical ripening and PTL in mice, which suggests that P4-mediated suppression of cervical ripening is critical for prevention of preterm birth (PTB) [18].

It is known that inflammation is detected in the reproductive tract of healthy women undergoing TL without infection [19]. The activation of inflammatory pathways precedes the onset of labor, both at term and preterm, initializing cervical effacement and dilation, and facilitating uterine contractions [20]. Importantly, the key underlying mechanisms are similar for TL and PTL [16]: once the maternal immune system is activated, whether it is at term (physiologic inflammation) or pre-term (pathologic infection), the inflammatory cascade begins in the uterus, promoting cervical ripening and myometrial contraction, culminating in delivery of the fetus [19]. In cervix, withdrawal of P4 initiates expression of pro-inflammatory cytokines and activation of hyaluronic acid synthase (HAS) [19,21,22]. The pro-inflammatory cytokines (i.e., IL-1α, IL-1β, IL-6, and TNFα) promote surrounding cells to secrete matrix metalloproteases (MMPs), causing the biodegradation of extracellular matrix (ECM) [19,23,24,25]. The presence of infection (bacterial or viral) can prematurely trigger the cervical ripening process, promoting preterm uterine contractions and PTB [19]. Thus, we hypothesized that in pregnant mice, inflammation (physiologic or infection-induced) causes cervical P4 withdrawal by inducing P4-metabolizing enzymes, which accelerates cervical ripening and labor onset (term or preterm). Our first aim is to examine the expression of both P4-metabolizing enzymes in mouse cervix throughout gestation during TL and LPS-induced PTL using immunohistochemistry and real-time qRT-PCR.

Preterm birth (the delivery before 37 weeks of gestation) affects 10.6% of human pregnancies worldwide [26,27]; its etiology is not fully understood [28]. Preterm neonates have 40% higher mortality and morbidity rates compared to term babies [29]. Existing therapies, mostly targeting uterine contractility, cannot reduce the incidence of PTB [30]. Selective Progesterone Receptor Modulator called promegestone (aka R5020) is a progestin, non-metabolizable by P4-metabolizing enzymes and a promising new drug for PTB prevention. In our recent study, R5020 was found capable of delaying TL and blocking PTL in mice [14]. In that study, we focused particularly on the inhibitory effect of R5020 on myometrial contractility and did not explore cervical changes. Here we hypothesized that R5020 may prevent infection-induced PTB in mice also by inhibiting premature cervical inflammation and accelerated cervical ripening. Our second aim is to investigate if R5020 can decrease premature cervical ripening in pregnant mice caused by systemic or local LPS administration, thus preventing PTB.

## 2. Materials and Methods

### 2.1. Animal Model

Animals were housed in a pathogen-free, humidity controlled 12 hr light, 12 hr dark cycle animal facility of The Centre for Phenogenomics (TCP, Toronto, Canada) with free access to food and water. Guidelines set by the Canadian Council for Animal Care were strictly followed in handling mice. Young (8–12 weeks old) virgin female CD-1 outbred mice were naturally bred; the morning of vaginal plug detection was designated as gestational day (GD)1. The ratio of male to female was 1:1. The gestational length in CD-1 mice is 19.5 days on average (range 19.1–19.9), and term delivery under these conditions occurred during the evening of GD19 or morning of GD20.

### 2.2. Experimental Design

Mouse model of term labor: Cervical samples were collected from non-pregnant (NP) and pregnant female CD-1 mice on GD10, 15, and 18. Animals were euthanized by carbon dioxide inhalation and tissue was collected at 10 a.m. on all days, with the exceptions of the TL samples that were collected during active labor once the animals delivered at least one pup on the evening of GD19 or morning of GD20. TNIL samples were collected on the evening of GD18 or early morning of GD19 before the start of TL. Tissue was collected from six animals (*n* = 6) per group for each experiment.

Mouse model of infectious PTL: Preterm labor was induced by systemic (A) or local (B) administration of lipopolysaccharide (LPS). Lyophilized LPS (serotype; O55:B5, Sigma, St Louis, MO) was dissolved in saline to a stock concentration of 1 μg/μL and administered on GD15, which corresponds to 75% of gestational length in mice, and is equal to 32 weeks of gestation in women.

(A) Model of systemic maternal infection: Pregnant CD-1 mice (*n* = 36) were injected intraperitoneally (IP) with LPS (50 μg in 100 μL) or vehicle (100 μL of sterile saline) on GD15. Animals were then observed hourly for signs of labor except during the interval from midnight to 6 am. IP injection of 50 μg LPS/mouse on GD15 causes PTB in 100% of animals in the “Vehicle + LPS” group within 24 h with minimal signs of maternal morbidity [14,31].

(B) Model of local intrauterine (IU) infection: Pregnant CD-1 mice (*n* = 56) were anesthetized on GD15 with isoflurane, then the lower abdominal area was shaved and antiseptically prepared using 70% alcohol and iodine solution. A laparotomic surgery was performed on each mouse and the right uterine horn was exposed through the incision. LPS (125 μg of LPS in 100 μL) or vehicle (100 μL of sterile saline) was administered via IU infusion in between two lower gestational sacs. To end the procedure, the fascia was closed with continuous vicryl sutures, and the skin was closed using 7 mm stainless steel wound clips. Mice were given a subcutaneous injection of 0.1 mL of 0.03 mg/mL buprenorphine and 0.5 mL sterile saline post-surgery for pain management and to prevent dehydration. The 125 μg dose of IU LPS was consistently proven to induce preterm delivery between 14 and 24 h post-surgery, while maintaining low maternal morbidity [31,32].

### 2.3. R5020 Administration

On GD14, pregnant mice were randomly divided into two groups; half of all animals were subcutaneously (SC) injected with R5020 (0.2 mg/dam in ethanol/corn oil), and the other half received SC injection of vehicle (ethanol/corn oil). Next, 24 h from the R5020/vehicle (saline) administration, mice received a second injection of R5020/vehicle (saline), respectively. On GD15, half of the R5020-treated animals received LPS, and the other half received vehicle (saline) injections; similarly, half of the vehicle (ethanol/corn oil)-treated animals received LPS, and the other half received vehicle (saline) injections. On GD16, animals that did not deliver preterm received a third injection of R5020/vehicle (saline). The scheme of treatments and collections is shown in Figure S2.

### 2.4. Tissue Collection

To assess the effect of R5020, animals (*n* = 6−9 per group) were euthanized 6–hours, 18 h, and 24 h post-LPS administration (Figure S2). Mouse cervical tissues were dissected with careful precision to not include uterine and vaginal tissues, as well as the bladder to only excise the “cervix proper”.

### 2.5. Real-Time Polymerase Chain Reaction (PCR) Analysis

Total mRNA was extracted from mouse cervical tissue using Trizol (Gibco, Canada) according to the manufacturer’s instructions. To remove genomic DNA contamination, the samples were treated with 2.5 μL DNase I (Qiagen, Hilden, Germany) for 20 min, and then purified by using the RNeasy MiniElute Cleanup Kit (Qiagen, Hilden, Germany). RNA concentration was measured using the NanoDrop 1000 spectrophotometer. qRT-PCR was performed with the CFX-384 TouchTM Real-Time PCR Detection System using SYBR Green chemistry. SYBR Green RT mix contains oligo dT primers. Transcript levels were analyzed across gestation, TL, and during PTL. Primers used in this study are listed in Table S1. Each sample was set up in technical triplicates. The Ct value represents the number of cycles, or the extent of cDNA amplification, needed to detect a fluorescent signal (SYBR green) that exceeds background noise. Data points above a Ct value of 33 were excluded as per standard practice. The relative gene expression of each sample was first determined by calculating the mean of the three Ct values using the manufacturer’s software (CFX Manager Version 3.1, Bio-Rad), and by applying a comparative Ct method (ΔΔCt method). A “no template control” (NTC) was used to control for contamination in the master mix. Results for mouse gestational profile (*n* = 6/GD) are shown as gene expression relative to mean of three reference genes: peptidyl-prolyl isomerase A (*Ppia*), TATA-Box binding protein (*Tbp*), and hypoxanthine phosphoribosyl transferase 1 (*Hprt1*). Results for IP- and IU-PTL models with R5020 treatment (*n* = 5–6/group) were normalized to reference genes *Tbp, Hprt,* and glyceraldehyde-3-phosphate dehydrogenase (*Gapdh*). Expression of genes of interest is shown as fold change relative to the “vehicle-sham” group.

### 2.6. Immunohistochemistry

Whole cervical tissue was fixed in 4% PFA, dehydrated by alcohol and xylene baths, paraffin-embedded, and sectioned in the cross-sectional plane at a thickness of 5 μm. Slides containing endocervical tissue were used for analysis. The slides were baked overnight at 37 °C, deparaffinized in xylene and rehydrated in grades of ethanol, then quenched with 3% H_2_O_2_ (prepared in methanol) to reduce endogenous peroxidase activity. Heat-induced antigen retrieval was performed using sodium citrate. Sections were then incubated with primary antibodies for SRD5A1 (ProteinTech, 26001-1AP, Chicago, IL, USA), 20α-HSD (aka AKR1C1) (GeneTex, GTX105620, Hercules, CA, USA), HABP (Amsbio, AMS.HKD-BC41, Abingdon, UK) proteins, and F4/80, a specific marker of mouse macrophages (Bio-Rad, MCA497GA, Hercules, CA, USA) or non-specific rabbit IgG (negative control, BioLegend, 400601, Hercules, CA, USA) overnight at 4 °C. On the next day, sections were washed and then incubated with a biotinylated secondary anti-rabbit or anti-rat antibody for one hour and with streptavidin–horseradish peroxidase solution (SA1007) for 30 min at room temperature with 3× washing in between. Sections were developed with a 3,30- diaminobenzidine (DAB) kit under a microscope. Slides were counterstained with hematoxylin (Gill’s No. 1 Accustain^®^, Edgewood, MD, USA), dehydrated, and mounted (Surgipath Micromount^®^ mounting media, Toronto, ON, Canada).

Immunofluorescence: Whole cervical tissue was processed as mentioned for immunohistochemistry. Post-antigen retrieval, slides were blocked with Sudan black to quench any endogenous fluorescence. Sections were then incubated with primary antibodies for P4 (Bio-Rad, 7720-17004, Hercules, CA, USA), PRA/B (ThermoFisher, MA5-14505, Danvers, MA, USA), PRB (Cell Signalling, 3157, Danvers, MA, USA), and rabbit IgG (BioLegend, 400601, Hercules, CA, USA) overnight at 4 °C. On the next day, sections were washed and then incubated with a fluorophore-conjugated secondary anti-rabbit antibody (Vector Labs, BA110, BA9400, Hercules, CA, USA). Slides were incubated with DAPI and mounted.

### 2.7. Image Analysis and Quantification

The murine endocervix was examined using the Olympus BX61 Motorized Microscope and MicroSuiteTM system (Olympus America Inc. Valley, PA, USA). All slides were scanned (Axio Scan.Z1 Slide Scanner, Carl Zeiss Microscopy, Jena, Germany), and the pictures were imported into Visiopharm NewCast Software (version 2018.9), EngineTM, and Viewer software modules for quantification. Analysis of the mouse endocervix region was performed. Endocervix was divided into a longitudinal muscle layer (LM) and a circular muscle layer (CM) as was reported earlier [4] (shown in Figure S3). Each region of interest (ROI) was masked using the Visiopharm software labelling tool (Figure S4). First, a standard Visiopharm application was used to exclude white space from within tissue. Next, a specialized Visiopharm application was used to identify DAB brown staining. There were three biological replicates for each GD in each study group (TL, PTL, and R5020-treated). The positively stained areas of the cervical tissue were visualized by the brown deposition. To achieve a blinded non-bias analysis, slide identities were replaced with a randomly assigned numerical alias before quantification. The relative amount of protein staining in each individual cervical tissue sample was calculated as the ratio of brown staining area to the total masked area of tissue (brown area to tissue area ratio, BTR). These data are shown as “percentage BTR”. These quantities were compared across normal mouse gestation and TL as well as two mouse models of PTL.

### 2.8. Statistical Analyses

Statistical analyses were performed using GraphPad Prism version 9.4.1 (GraphPad Software, Inc. Hercules, CA, USA). All data sets were analyzed using Grubb’s test using GraphPad software and significant outliers (*p* < 0.05) were excluded. Statistical analysis of real-time qRT-PCR data and quantitative imaging protein data from the TL model was performed using ordinary one-way ANOVA with the mean of each column being compared to the mean of every other column. For data pertaining to TL models, statistically significant differences (*p* < 0.05) are denoted by different letters. Statistical analysis of real-time qRT-PCR data and quantitative imaging protein data from the PTL models was performed using two-way ANOVA with multiple comparisons, comparing cell means regardless of rows and columns. For data pertaining to PTL models, statistically significant differences are marked by asterisks (* *p* < 0.05, ** *p* < 0.01, and *** *p* < 0.001).

## 3. Results

### 3.1. Term Labor Is Associated with Increased Expression of P4-Metabolizing Enzymes and Decreased Levels of P4 in Mouse Cervix

**Figure 1:**
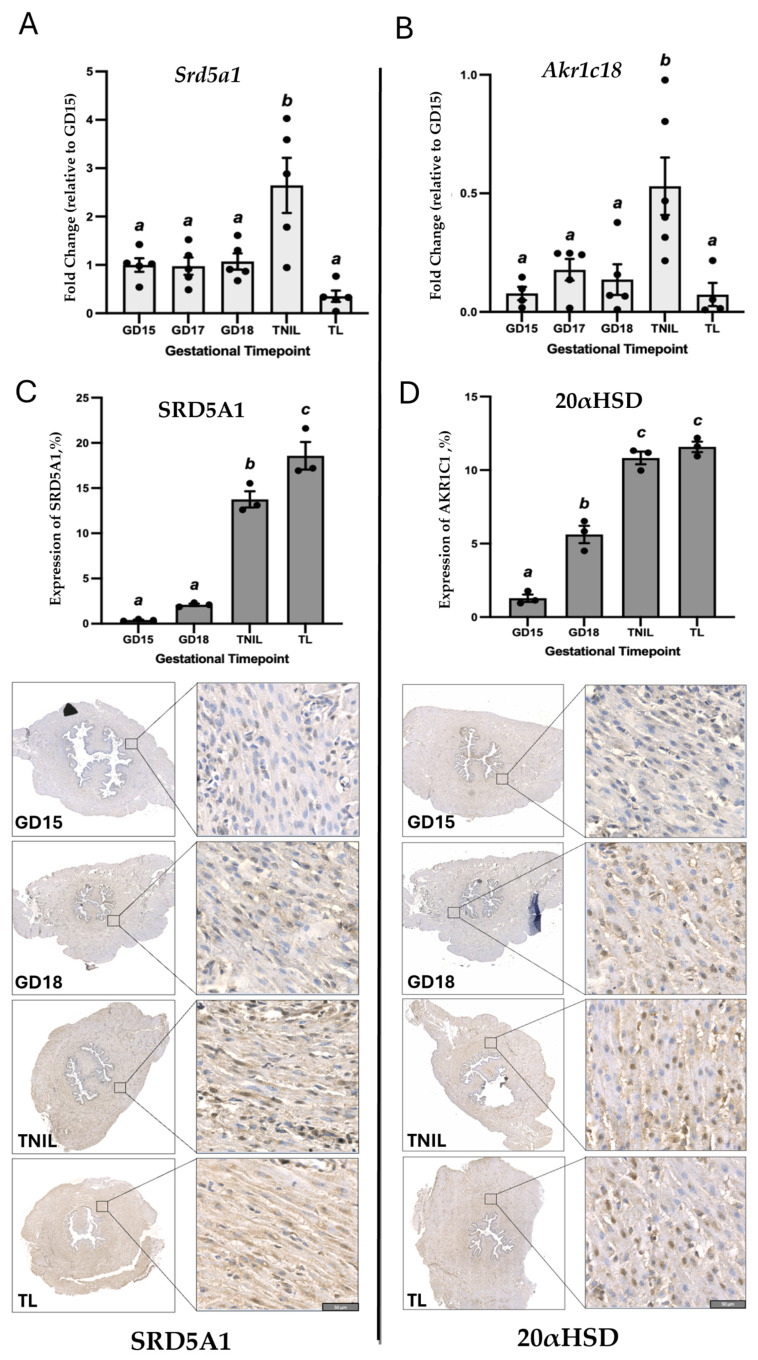
Protein and transcript levels of P4-metabolizing enzymes; SRD5A1 and 20α-HSD in pregnant mouse cervix throughout gestation and term labor (TL). (**A**,**B**) Expression levels of *Srd5a1* (**A**) and *Akr1c18* (**B**) mRNA transcripts (light grey bars) are shown as fold change relative to gestational day (GD)15 (*n* = 5–6/GD). Different letters denote statistical differences determined at *p* < 0.5. (**C**,**D**) Representative images and quantification of immunopositive SRD5A1 (**C**) and 20α-HSD (**D**) proteins (dark grey bars) in murine cervix by the Visiopharm software (version 2018.9) Engine^TM^. Shown are cross-sections of endocervix from pregnant (GD15, GD18, and GD19/term-not-in-labor (TNIL), and laboring (TL) mice immunostained with antibodies against SRD5A1 (**left**) and 20α-HSD proteins (**right**); *n* = 3/GD. Positive brown staining indicates presence of proteins. Blue shows hematoxylin counterstaining. Magnification is at 100×. Scale bar = 50 μm.

**Figure 2:**
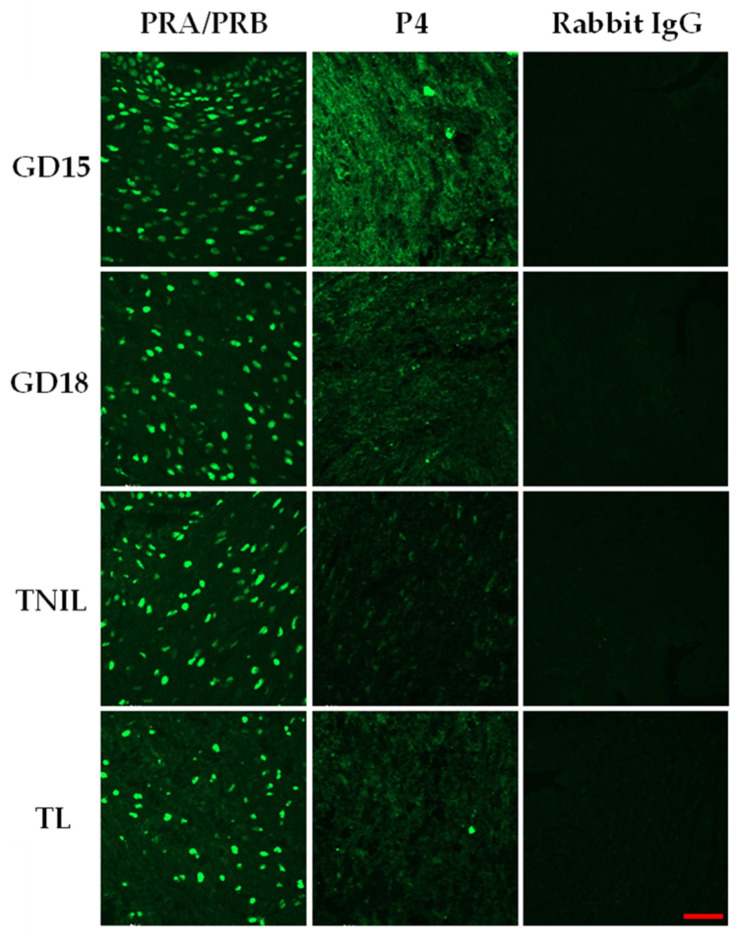
Progesterone receptors (PRs) and progesterone (P4) levels in pregnant mouse cervix. Representative immunofluorescence images of mouse cervical tissues stained with antibodies against total PRs (left panel) and P4 (middle panel); rabbit IgG was used as a negative control (right panel). Shown are cross-sections of endocervix from pregnant (GD15, GD18, and GD19/TNIL) and laboring (TL) mice. Green staining indicates presence of protein; *n* = 3. Scale bar = 50 μm.

Pregnant CD-1 mice were used as a model of normal gestation and TL. We first evaluated the proteins and mRNA levels of key P4-metabolizing enzymes SRD5A1 and 20α-HSD in cervical tissue. As determined by real-time qRT-PCR, transcript levels of both genes (*Srd5a1* and *Akr1c18)* were relatively stable throughout mouse gestation, with a significant increase before labor at gestational day (GD)19 as compared to late gestation (GD15-18, *p* < 0.05, Figure 1A,B). Immunostaining of both 20α-HSD and SRD5A1 proteins was detected in murine endocervix, increasing towards term (Figure 1C,D). Spatially, both SRD5A1 and 20α-HSD proteins were predominantly cytoplasmic, localized within the perinuclear region of uterine SMCs. The temporal protein expression pattern for both enzymes was similar to gene expression: SRD5A1 increased significantly at GD19/TNIL as compared to GD15-18 (*p* < 0.05); and peaked during TL (*p* < 0.05) (Figure 1C). The 20α-HSD protein expression was significantly increased at GD19/TNIL and TL (*p* < 0.05) (Figure 1D). The levels of tissue P4 and PRs in the mouse endocervix were evaluated throughout gestion using immunofluorescence. The PR-A/PR-B, protein levels were relatively consistent throughout gestation and TL (Figure 2, while the P4 level was high on GD15 and gradually declined towards TL (Figure 2).

**Figure 3:**
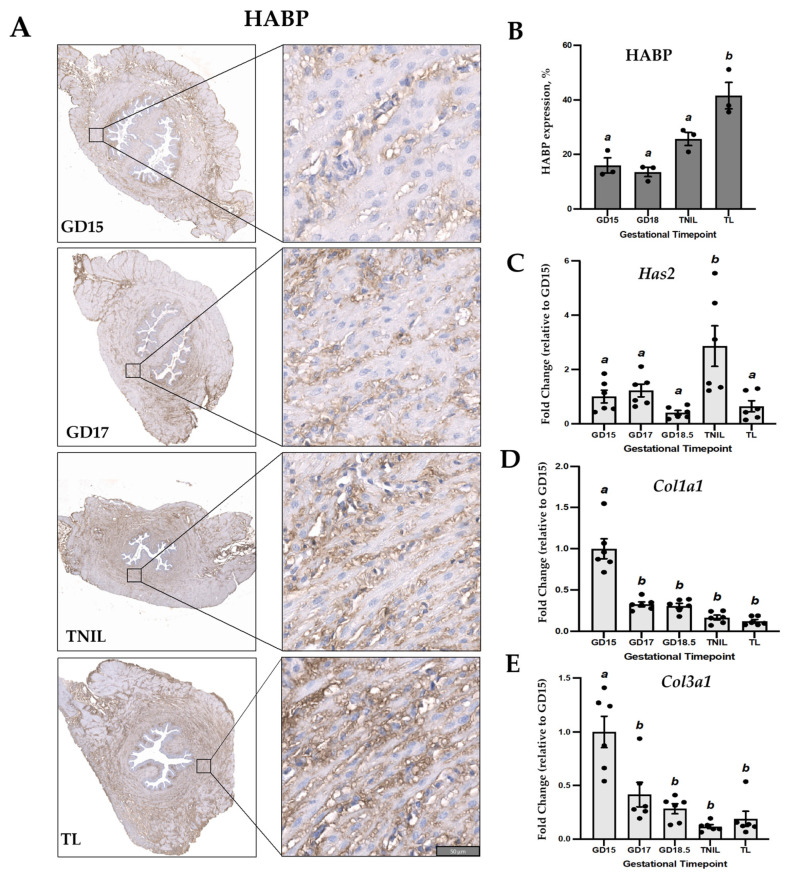
Markers of cervical ripening in pregnant mouse cervix throughout gestation and term labor. (**A**) Representative images of immunohistochemical localization of HA binding protein (HABP) in the endocervix of mice on gestational day (GD)15, GD18, term-not-in-labor (TNIL)/GD19, and term labor (TL). Brown staining indicates the presence of HABP. Scale bar = 50 μm. (**B**) Protein levels of immunoreactive HABP were quantified by the Visiopharm software (version 2018.9) Engine^TM^. Bar graphs (dark grey) show protein levels expressed as a percentage brown to total masked tissue ratio (BTR); *n* = 3. (**C**–**E**) Transcript levels of (**C**) *Has2,* (**D**) *Col1a1,* and (**E**) *Col3a1* in mouse cervix during gestation and TL (light grey bars). Graphs show gestational changes (fold change relative to GD15, *n* = 5–6/GD). Statistical significance was determined by ordinary one-way ANOVA with multiple comparisons. Data shown as mean ± SEM. Different letters denote statistical difference at *p* < 0.05.

Markers of cervical ripening were also assessed throughout gestation. HA binding protein (HABP), which binds to the mature HA in the extracellular matrix providing tissue hydration, was used as an indirect measure of HA levels in the mouse endocervix. Spatially, cervical HABP was predominantly extracellular, and was detectable throughout gestation and TL (Figure 3A). Quantitative assessment showed progressive increase in HABP levels in the mouse endocervix towards TL as compared to GD15, GD18, and TNIL (*p* < 0.05) (Figure 3B). Major HA synthase enzyme is encoded by the *Has2* gene. As determined by real-time qRT-PCR, *Has2* transcript levels in mouse cervix were relatively stable throughout gestation and TL, with a sharp 3-fold increase at term (TNIL, Figure 3C). Vice versa, major ECM fibrillar proteins, collagen type 1 and collagen type 3, which are known to provide tensile strength to the uterine cervix, notably decreased towards term: mRNA levels of both Col1a1 (Figure 3D) and Col3a1 (Figure 3E) were significantly (*p* < 0.05) higher on GD15 as compared to GD17, GD18, GD19/TNIL, and TL. Increase in cervical ripening proteins was associated with a massive infiltration of F4/80 positive macrophages in mouse endocervix (Supplementary Figure S1). F4/80 (aka EMR1: EGF-like module-containing mucin-like hormone receptor-like 1) is a protein encoded by the adhesion G protein-coupled receptor E1 (*ADGRE1*) gene. It is a well-recognized marker of mouse macrophages.

### 3.2. LPS-Mediated Inflammation Induces Cervical Expression of P4 Metabolizing Enzymes, P4 Decline, and Cervical Ripening Pre-Term

**Figure 4:**
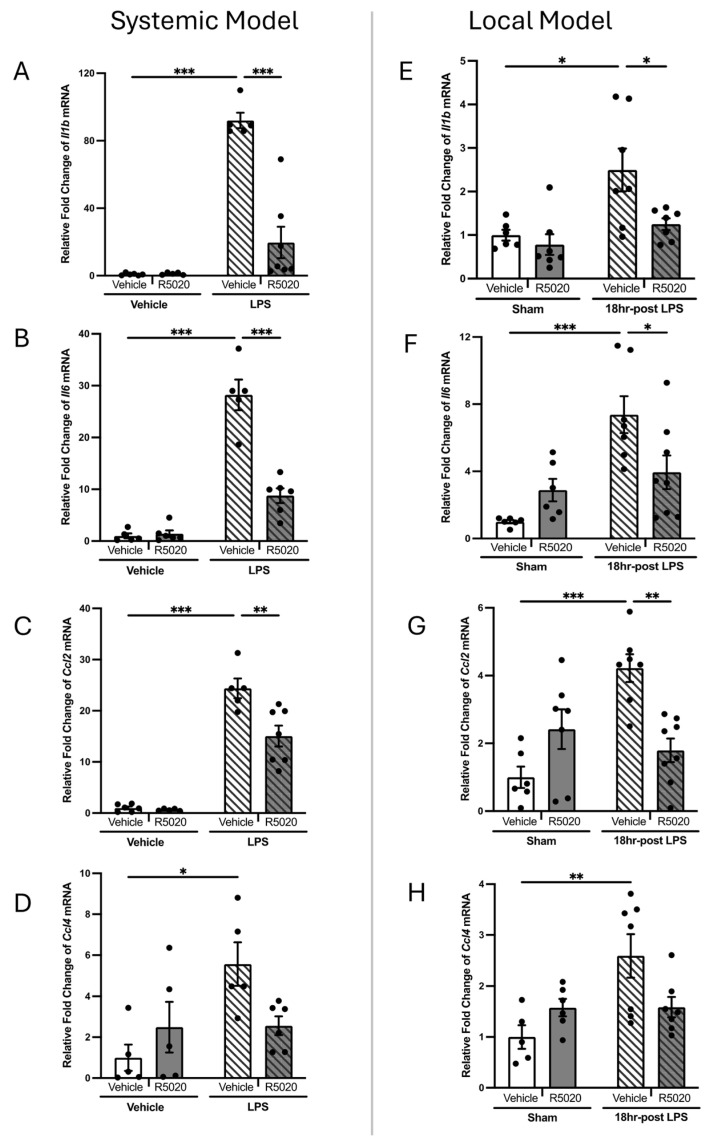
Transcript levels of pro-inflammatory cytokines and chemokines in pregnant mouse cervix during PTL. Shown are mRNA levels of *Il1b, Il6, Ccl2,* and *Ccl4* cytokines in murine cervix from GD16 pregnant and preterm laboring mice induced by LPS: systemic maternal inflammation (left–systemic PTL Model: (**A**–**D**)) and local uterine inflammation (right–local PTL Model: (**E**–**H**)). (**A**,**E**) *Il1b;* (**B**,**F**) *Il6,* (**C**,**G**) *Ccl2*, and (**D**,**H**) *Ccl4.* White bars represent control vehicle-treated groups, dark grey bars represent R5020-treated groups. Diagonally stripped bars represent LPS-induced groups. mRNA levels are shown as fold change relative to “vehicle” control in the systemic model (left) and to “sham” control in the local model (right). Data shown as mean ± SEM. Statistical analysis was determined using two-way ANOVA with multiple comparisons (*n* = 5–6/group). Statistically significant differences in mRNA expression levels between groups are marked by asterisks (* *p* < 0.05, ** *p* < 0.01, and *** *p* < 0.001).

**Figure 5:**
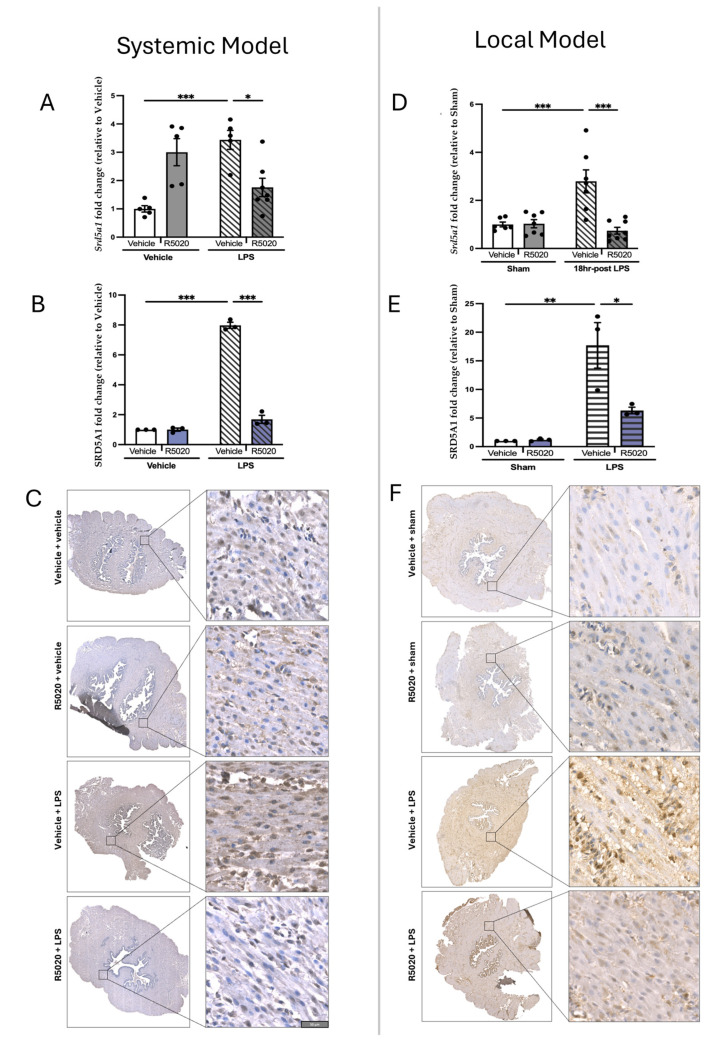

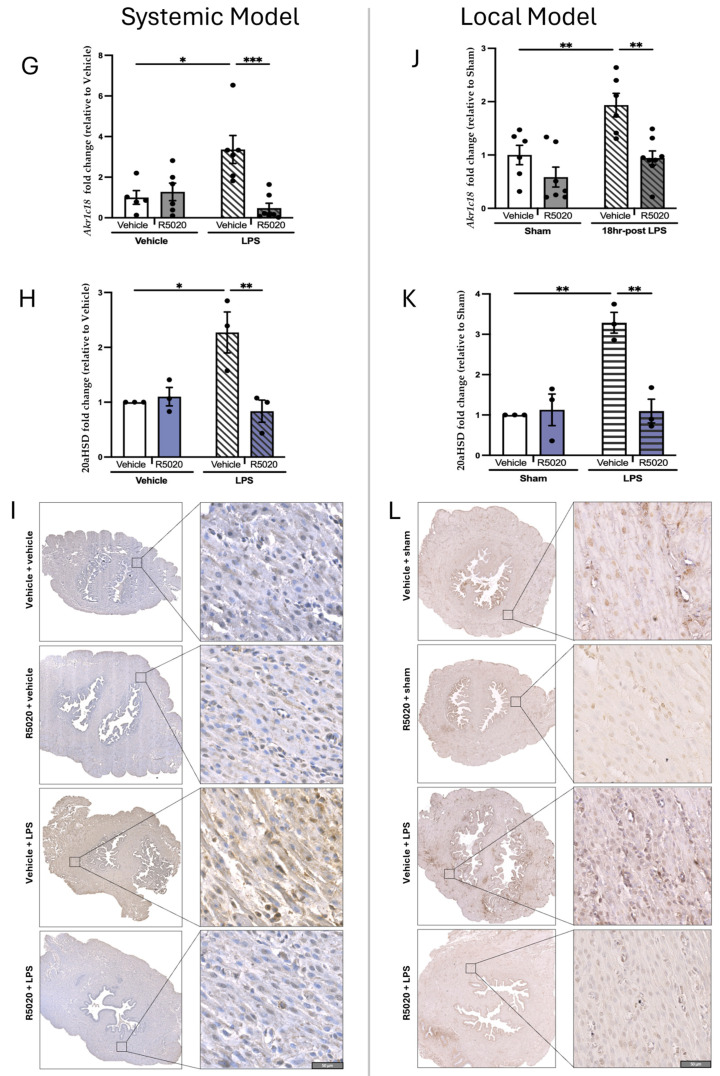
Protein and transcript levels of P4-metabolizing enzymes SRD5A1 and 20α-HSD in pregnant mouse cervix during LPS-induced PTL. Shown are mRNA levels of *Srd5a1* and *Akr1c18,* as well as SRD5A1 and 20αHSD protein expression levels in murine cervix from GD16 pregnant and preterm laboring mice induced by LPS: systemic maternal inflammation (left–systemic PTL Model: (**A**–**C**,**G**–**I**)) and local uterine inflammation (right–local PTL Model: (**D**–**F**,**J**–**L**)). White bars represent control vehicle-treated groups, and dark grey and blue bars represent R5020-treated groups. Stripped bars represent LPS-induced groups. mRNA levels of *Srd5a1* (**A**,**D**) and *Akr1c18* (**G**,**J**) are shown in dark grey bars as fold change relative to “vehicle” control in the systemic model (left) and to the “sham” control in the local model (right). Representative images of SRD5A1 (**C**,**F**) and 20αHSD (**I**,**L**) and quantification of immunopositive brown staining for SRD5A1 (**B**,**E**) and 20α-HSD (**H**,**K**) proteins (blue bars) in the mouse endocervix during PTL was performed by image analysis using Visiopharm software (version 2018.9) Engine^TM^; *n* = 3. Scale = 50 μm. Protein levels of SRD5A1 (**B**,**E**) and 20αHSD (**H**,**K**) are shown as fold change of brown staining area to total masked tissue area ratio (BTR). Statistical analysis was performed using two-way ANOVA followed by Bonferroni post-test with multiple comparisons. Data are shown as mean ± SEM. Statistically significant differences in expression levels between experimental groups are marked by asterisks (* *p* < 0.05, ** *p* < 0.01, and *** *p* < 0.001).

**Figure 6:**
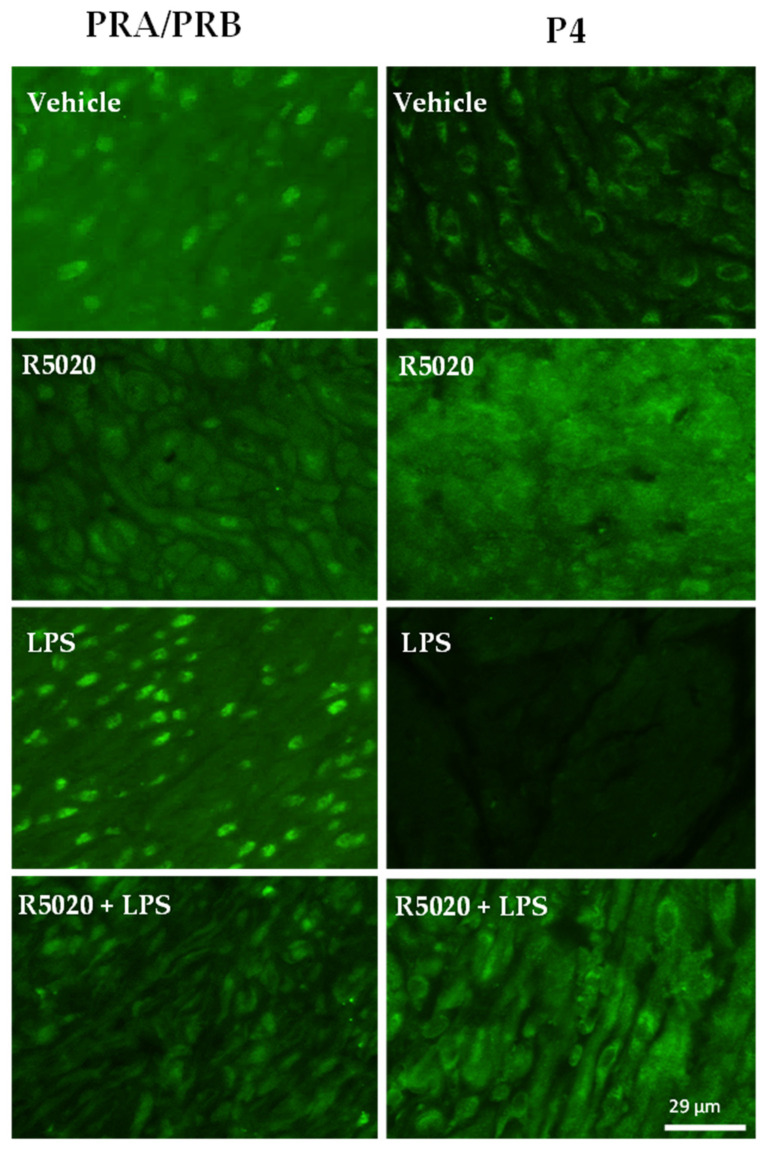
Progesterone receptors (PRs) and progesterone (P4) levels in pregnant mouse cervix during PTL induced by systemic maternal inflammation. Representative immunofluorescence images of mouse cervical tissues stained with antibodies against total PRs (left panel) and P4 (right panel). Shown are cross-sections of endocervix from GD16 pregnant mice (control vehicle-treated, control R5020-treated), R5020-treated/LPS-induced pregnant not-in-labor, and LPS-induced preterm laboring mice. Green staining indicates presence of protein; *n* = 3. Scale bar = 29 μm. Negative control—Rabbit IgG (see Figure 2).

Two mouse models of LPS-induced PTB were used in this study: systemic inflammation (achieved through intraperitoneal injection of LPS) [14,31] and local uterine infection (achieved through intrauterine infusion of LPS) [14,31]. In both cases, PTB occurred within 24 h post-injection. A significant increase in mRNA levels of inflammatory markers; *Il1b*, *Il6*, *Ccl2*, and *Ccl4* (*p* < 0.05) was detected in mouse cervix 6 h after intraperitoneal injection (Figure 4A–D) and 18 h after intrauterine infusion of LPS (Figure 4E–H) as compared to the control vehicle-treated animals. Irrespective of the mode of LPS administration, local and systemic inflammation significantly increased SRD5A1 (Figure 5A–F) and (Figure 5G–L) protein and transcript levels on GD16, 6–24 h post-injection. This premature activation of P4-metabolizing enzymes was reflected in premature decline in P4 in cervical tissue on GD16 compared to vehicle-injected controls (Figure 6, right panel), with an increase in PR levels (Figure 6, left panel).

**Figure 7:**
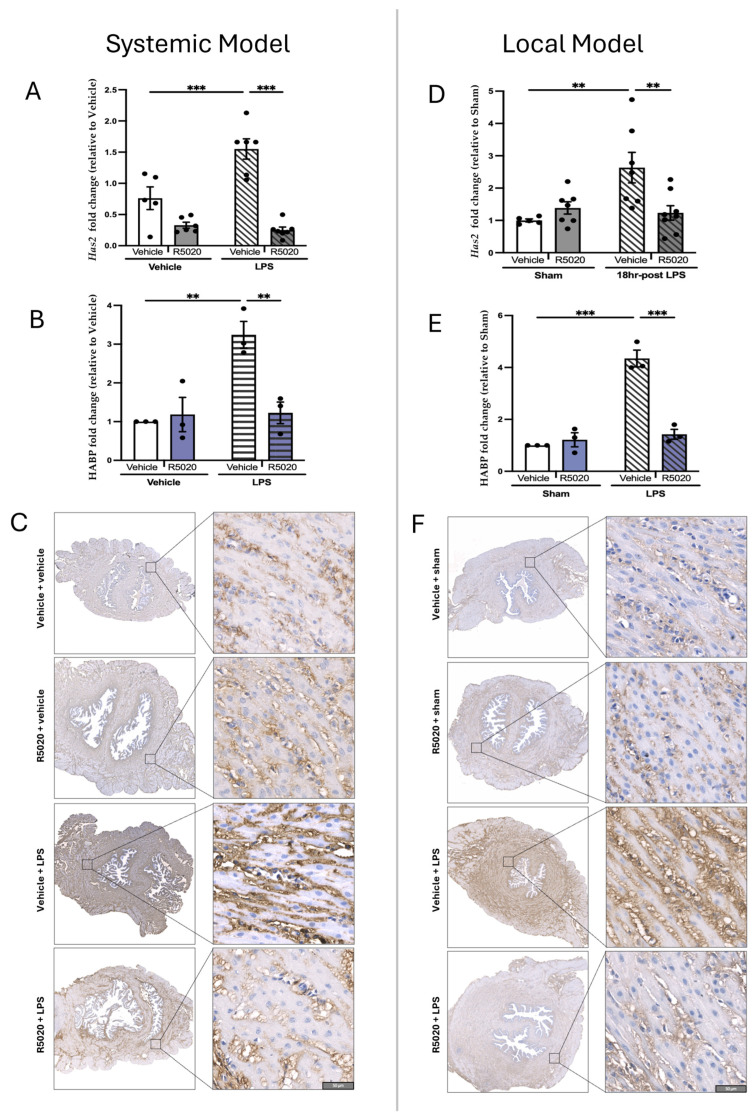
Transcript levels of *Has2* and protein levels of HABP in pregnant mouse cervix during LPS-induced PTL. (**A**) Transcript levels of *Has2* are shown as fold change relative to “vehicle” (left–systemic PTL model) or (**D**) relative to “sham” (right–local PTL model). Data shown as mean ± SEM (*n* = 5–6/group). (**C**,**F**) Representative images of immunohistochemical localization of HABP in the mouse endocervix during PTL and pregnant GD16 controls. Brown staining indicates presence of protein. (**B**,**E**) Bar graphs show protein levels of HABP expressed as fold change of %, brown to total masked tissue ratio (BTR) relative to “vehicle” (left) or “sham” (right) groups; *n* = 3. Quantification of HABP staining was performed by image analysis using Visiopharm software (version 2018.9) Engine^TM^. White bars represent control vehicle-treated groups, dark grey or blue bars represent R5020-treated groups. Stripped bars represent LPS-induced groups. Statistical analysis was performed using two-way ANOVA followed by Bonferroni post-test with multiple comparisons. Statistically significant differences in HABP expression levels between time points are marked by asterisks (** *p* < 0.01, and *** *p* < 0.001). Scale bar = 50 μm.

Premature cervical ripening was observed in both LPS models. A significant induction of *Has2* transcript levels was observed 6 h after systemic LPS administration (Figure 7A, *p* < 0.001) and 18 h after intrauterine LPS infusion (*p* < 0.01, Figure 7D) as compared to vehicle-treated controls. The staining intensity of HABP was dramatically increased in cervical tissues of mice treated with LPS as compared to control animals (Figure 7C,F). Quantitative image analysis showed a significant (*p* < 0.01) increase in HABP protein level in mouse cervical tissues after systemic (Figure 7B) and local LPS administration (Figure 7E).

### 3.3. R5020 Blocks LPS-Induced Inflammation, Cervical Ripening and Preterm Labor

Prophylactic administration of R5020 (0.2 mg/dam) 24 h prior to LPS prevented PTB in 100% of pregnant mice irrespective of the route of LPS administration. In animals pre-treated with R5020 before systemic or local LPS induction (“LPS + R5020 groups”), the transcript levels of inflammatory markers *Il1b*, *Il6,* and *Ccl2* were significantly lower (*p* < 0.05) than in the “LPS only” group (Figure 4A–C,E–G). *Ccl4* remained unaffected by the R5020 prophylactic treatment in both models (Figure 4D,H). Importantly, R5020 pre-treatment did not affect the basal cytokine mRNA levels in the vehicle and sham groups. The transcript levels of cervical ripening marker *Has2* in animals pre-treated with R5020 before LPS administration were maintained low and comparable to control vehicle-injected mice (Figure 7A,B). Similarly, the LPS-mediated increase in HABP protein was prevented by R5020 (Figure 7C,F). Notably, prophylactic treatment of pregnant mice with R5020 before LPS administration (systemic or local) also prevented the inflammation-induced upregulation of P4-metabolizing enzymes SRD5A1 (Figure 5A–F) and 20α-HSD (Figure 5G–L). Immunostaining of SRD5A1 (Figure 5C,F) and 20α-HSD (Figure 5I,L) in the endocervix of a R5020-treated mouse was no different from the control vehicle-treated animals. In addition, R5020 prevented the inflammation-induced increase in PR levels and premature decline in P4 caused by LPS in cervical tissue (Figure 6).

## 4. Discussion

Multiple lines of evidence suggest that local withdrawal of P4 occurs in the uterus in preparation for labor onset [33]. The role of two P4-metabolizing enzymes 20α-HSD and SRD5A1 varies in different uterine compartments. For instance, in the uterine muscle, myometrium, activity of the 20α-HSD enzyme was found to play a major role in the physiologic P4 withdrawal near term [14]. Our research [8,34] and that of others [15] suggests that increased abundance and activity of the 20α-HSD enzyme in myometrium during TL and PTL is induced by pro-inflammatory mediators which results in P4 withdrawal. The bacterial endotoxin LPS mimicking pathologic infection upregulates 20α-HSD expression in-vivo in mouse myometrium and in-vitro in human myocytes [34]. On the other hand, the role of 20α-HSD in the uterine cervix currently is not fully understood. Multiple studies focused mostly on the SRD5A1 in the mouse cervix, as its knockout prevented cervical remodeling and delayed parturition in mice [35]. The mouse model of 20α-HSD knockout similarly presented with delayed labor [36], which suggests that 20α-HSD plays a critical role in normal parturition. In a current study, we examined the effect of physiologic and pathologic inflammation on the cervical expression of P4-metabolizing enzymes in pregnant mice. We found that both 20α-HSD and SRD5A1 were expressed in mouse cervix, and their levels significantly increased at term and during TL (Figure 1). Labor in humans and rodents is associated with secretion of inflammatory mediators [37,38] and subsequent infiltration of immune cells in uterine tissues, including myometrium [38] and cervix [39]. During the ripening process preceding TL, physiological inflammation is detected in the mouse cervical stroma, and characterized by an increased density of resident leukocytes, particularly macrophages [40]. Macrophages mediate a crosstalk between stromal fibroblasts and smooth muscle cells to regulate local responsiveness to P4 [19]. Proteolytic activity of macrophages is responsible for ECM remodeling and degradation [41], which provides a molecular basis for increased biomechanical compliance of cervical tissue in preparation for labor onset. The current data directly confirms that an upregulation of P4-metabolozing enzymes and a consequent local decline in P4 (Figure 2) were associated with increased cervical inflammation, depicted in the massive infiltration of F4/80-positive mouse macrophages (Figure S1) and weakening of ECM in the murine endocervix before and during TL. Local P4 withdrawal, manifested by decreased P4 immunofluorescence levels in pregnant mouse endocervix at term (TNIL and TL vs. GD15), was not associated with changes in PRA/B isoforms (Figure 2). Our data support earlier results reporting that PRs in cervical stromal cells are consistent throughout gestation during labor and postpartum [10].

In an earlier study by Yellon et al., the role of P4 withdrawal was shown in prostaglandin receptor-deficient (Ptgfr−/− KO) mice, where luteolysis was prevented, resulting in persistent P4 production and TL blockade [42]. Importantly, when Ptgfr (−/−) mice underwent the procedure of ovaries removal, there was a decline in P4, leading to parturition. These authors pointed to the link between P4 withdrawal, inflammation, and cervical ripening, as macrophage density in cervical tissues of term-pregnant KO mice not capable to deliver due to high P4 levels was 3-times lower compared to non-pregnant KO females; these mice lack cervical ripening as collagen degradation was positively correlated with macrophage numbers [42]. Collagen biodegradation is a part of the cervical softening and ripening process in preparation for term parturition. We found a significant decrease in both major collagen type 1 and type 3 *Col1a1* and *Col3a1* transcripts in mouse cervix after GD15 (Figure 3), which indicates that fibrillar collagen synthesis is also reduced in preparation for TL. These results fully support numerous published data which state that timely changes in cervical ECM (both processes, a decrease in collagen biosynthesis and an increase in biodegradation) are crucial for cervical remodeling in preparation for labor onset.

In addition, murine cervix is a dense connective tissue; the remodeling process prior to labor depends on collagen turnover, biodegradation, and disorganization mediated by HA. In mice, cervical ripening is initiated 2–4 days before birth (i.e., GD15-16), when systemic P4 is at its peak [16]. HA synthase isoenzymes (encoded in mice by *Has1-3* genes) were found in the mouse cervix during gestation, with *Has2* expression increased on GD18 [43]. There is evidence for *Has2* expression in fibroblasts and smooth muscle cells, both components of mouse cervical stroma [44]. We recently reported that HA binding protein/HABP expression, indicative of the HA levels, is low in a non-pregnant murine cervix, but significantly increased with advanced gestation in both the endo and ectocervix, peaking at term [4]. In situ quantification of HA in tissue sections is a sensitive tool to analyze its distribution during gradual cervical remodeling [45], pointing to the increased tissue hydration during TL. Our previous MRI data also showed a loss of organized endocervical structure in pregnant mice, providing means for cervical ripening [4]. Collectively, our earlier and current results suggest that cervical ripening during TL is an inflammatory process regulated by P4 metabolism, with the endocervix becoming well hydrated, disorganized, and less rigid, which creates a flexible structure able to stretch and dilate, allowing the fetus to pass during forceful labor contractions.

Next, we used two different models of PTL mimicking systemic and local infection and found that both P4-metabolizing enzymes were significantly upregulated in the mouse cervix by bacterial endotoxin LPS produced by Gram-negative bacteria (Figure 4, Figure 5, Figure 6 and Figure 7). LPS triggers the inflammatory cascade through Toll-like receptor 4 (TLR4), a member of a family of “pattern recognition receptors”. We have shown before that LPS can induce PTB causing inflammation in maternal (plasma, myometrium, and cervix) and fetal (placenta, amniotic fluid) tissues [14]. In this study, we confirm that both the *Has2* gene and HABP protein were significantly induced in mouse cervix by systemic [14] and local inflammation (Figure 7). Importantly, we found that this was associated with increased inflammatory markers, elevated cervical expression of 20α-HSD and SRD5A1, and local cervical decline in P4 during PTL (Figure 6). This can be explained both by the increased activity of P4-metabolizing enzymes and by the fact that LPS administration induces a significant decrease in systemic P4 levels. We noticed a similarity in cervical expression of P4-metabolizing enzymes between TL and PTL, which confirms their pivotal role in local P4 withdrawal in the cervix and labor onset. It has been previously reported that systemic P4 levels in serum fall during PTL induced by systemic or local bacterial product-induced infection [46,47,48]. P4 has potent anti-inflammatory properties [32,49,50] and is essential for protecting against LPS-induced pregnancy loss in mice [51].

Previously, we reported that in vivo prophylactic administration of promegestone (aka R5020), non-metabolizable by 20α-HSD, prevented LPS-induced PTB in mice by inhibiting myometrial activation [14]. Promegestone is a progestin, sold under the brand name Surgestone in a few countries, a medication used in menopausal hormone therapy and in the treatment of gynecological conditions caused by luteal insufficiency, including premenopausal disorders, and dysmenorrhea [52,53]. Promegestone tablets have a contraceptive effect and are used as a form of progestogen-only birth control [54].

Importantly, our current data show that R5020 also decreased cervical inflammation and remodeling (Figure 4, Figure 5, Figure 6 and Figure 7). We found that R5020 exerts anti-inflammatory effects by significantly decreasing LPS-induced transcript levels of major pro-inflammatory cytokines *Il1b* and *Il6* and chemokines *Ccl4* and *Ccl2* in the mouse cervix (Figure 4). These data directly correlate with our previous results indicating that R5020 significantly inhibited mRNA levels of cytokines (*Il1a, Il6*) and chemokines (*Ccl2, Ccl3, Ccl4, Cxcl1,* and *Cxcl2*) in the mouse myometrium and partially inhibited decidual and placental inflammation [14]. Importantly, R5020 significantly decreased cervical expression of the *Has2* gene and endocervical expression of HABP in LPS-induced pregnant dams (Figure 7). HA-mediated tissue hydration is one of the most critical events during cervical ripening, thus, inhibition of this process may represent an essential mechanism by which R5020 blocks labor. In other words, current results complement data on a blockade of myometrial contractility [14], and show that R5020 can directly inhibit cervical inflammation (Figure 4 and Figure 5) and ECM remodeling in murine cervix, which together prevent the onset of LPS-induced PTB.

The inhibition of pro-contractile and pro-inflammatory proteins in myometrium by R5020 is mediated through PRs [14]. We reported previously that the induction of labor in mice and humans is associated with a release of PRs from binding to the hormone in myometrial cells, which was a result of reduced cellular P4 levels [8,55]. We propose that, similar to myometrium, the main mechanism of R5020 action in the cervix is the maintenance of P4 signaling through strong binding to PRs. Research demonstrates the effectiveness of P4 in promoting cervical ripening, supported by studies involving PR antagonists and non-metabolizable progestins such as R5020 [56]. Furthermore, the review of existing literature on R5020 emphasizes its impact on PR turnover. R5020 is recognized as a strong agonist of PRs and has been shown to induce PR phosphorylation and degradation [57]. The observed reduction in PR levels following R5020 treatment (Figure 6, left panel) is expected and affirms the progestin’s action in the cervix. We reported that the strength and duration of the progestogenic effect of R5020 in suppressing LPS-induced myometrial activation is much higher than P4, which is metabolizable by 20α-HSD [14]. Notably, when compared the prophylactic effect of R5020 versus P4, we found that P4 administration only partially inhibited myometrial activation, while R5020 was able to completely block LPS-induced parturition and delay TL in mice [14]. These findings are consistent with a study by Hirsch et al., which showed that P4 treatment was unable to prevent PTB in pregnant mice infected with E. coli [47].

In humans, R5020 is metabolized by 21-hydroxylation to trimegestone (TMG), which has an even higher affinity and specificity to PRs than R5020 [58,59]. TMG itself is metabolized into 1β- and 6β-hydroxy-TMG, which possesses higher specificity to PRs [60]. Importantly, the half-life of TMG is 15 h, a few times longer than R5020 [52,61]. It was shown that TMG can suppress TL in rats, when administered topically, vaginally, or subcutaneously at late gestation [60,62]. Moreover, Kirby et al. showed that R5020 supported pregnancy in OVX mice until full term [63]. Similarly, Kuon et al. compared the effect of R5020 administration to P4 (17αOHP) on uterine contractility and cervical ripening during PTB in mice [64]. This study showed that 17αOHP failed to inhibit preterm delivery, while subcutaneous and vaginal R5020 administration completely blocked it. Taken together, R5020 or its derivatives, not metabolizable by 20α-HSD and SRD5A1, likely can maintain PR-mediated inhibition of pro-contractile and pro-inflammatory proteins in myometrium [14] and cervix, which may explain their great efficacy in the prevention of PTB induced by systemic or local infection in mice.

## 5. Study Limitations

There are several limitations to this study, including the model of PTB. The animal models of infectious PTB used here mimic systemic maternal infection and bacterial-induced intra-amniotic inflammation in pregnant women. Recently, new mouse models of human PTB caused by “sterile intra-amniotic inflammation” were developed, which mimic idiopathic spontaneous PTL. The PTB was triggered by endogenous “alarmin” molecules (HMGB1, IL1α, and S100) found in the amniotic fluid during term and preterm labor [65,66], which are derived from damaged and necrotic cells. These PTB mouse models may provide a future avenue into investigating the effect of R5020 on premature cervical ripening during idiopathic human PTL. In addition, s recent transcriptome and proteome study of mouse cervical remodeling by Nallasamy et al. discovered key genes and processes associated with pregnancy and labor [67]. Their publicly available database offers a wealth of high-throughput data and points to possible application of these modern methodologies for in-depth study of the molecular effects of R5020. More research on the preventive action of R5020 on cervical ripening using RNA-sequencing and proteome via mass-spectrometry is warranted. These future studies will expand our knowledge on the biological markers and molecular pathways affected by promegestone in reproductive tissues.

## 6. Conclusions

There is abundant evidence to support the notion that healthy parturition is an inflammatory process. We discovered that inflammation of mouse cervix, regardless of its origin (physiologic during TL, or pathologic during PTL), induced the expression of two main P4-degrading enzymes, SRD5A1 and 20αHSD. Our data suggest that these enzymes mediate local P4-withdrawal in the murine cervix during term and preterm labor.

We report here that the prophylactic administration of SPRM called promegestone, aka R5020, inhibited inflammation-induced P4-metabolism and cervical ripening, thus completely suppressing PTB in mice induced by systemic and local uterine inflammation. Promegestone binds to PRs with high affinity and is not metabolized by SRD5A1 or 20αHSD. A positive result of our animal studies using non-metabolizable progestin promegestone will inform discussion about the potential use of this or similar drugs for the prevention of PTL in in women at high risk of PTB. If the success of promegestone in animal models is replicated in humans, it may represent a new class of potentially safe and effective pro-gestational therapeutics for PTB prevention.

## Data Availability

The original contributions presented in this study are included in the article/Supplementary Materials. Further inquiries can be directed to the corresponding author.

## Supplementary Materials

The following supporting information can be downloaded at: https://www.mdpi.com/article/10.3390/cells14040242/s1, Figure S1: Immunohistochemical localization of macrophages in pregnant mouse endocervix throughout gestation and during TL; Figure S2: The scheme of drugs administration and sample collections in PTL mouse models; Figure S3: Smooth muscle layers in the transverse section of the murine endocervix; Figure S4: Representative images of longitudinal and circular smooth muscle layers of murine endocervix analyzed by Visiopharm software (version 2018.9) Engine^TM^; Table S1: Primer pair information for mouse genes examined using qRT-PCR.
