# Supplementary material for: Promegestone Prevents Lipopolysaccharide-Induced Cervical Remodeling in Pregnant Mice
Source: Cells. 2025 Feb 7;14(4):242. doi: 10.3390/cells14040242 (PMC11853409; doi:10.3390/cells14040242)
Supplement: Supplementary file 1 [file cells-14-00242-s001.zip › cells-3459253-supplementary.pdf]

cells MDPI

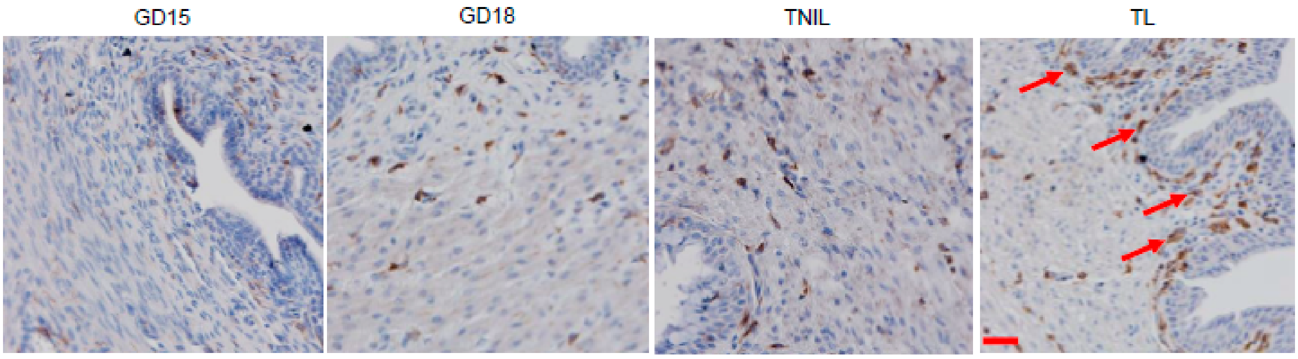

Figure S1. Immunohistochemical localization of macrophages in pregnant mouse endocervix throughout gestation and during TL. Macrophages were identified as F4/80 positive cells in the endocervix of pregnant (GD15, GD18, GD19/TNIL) and labouring (TL) mice, n=3. (stained dark brown, red arrows). Negative control - Rabbit IgG used at the same concentration as primary antibody. Scale bar = 50 µm.

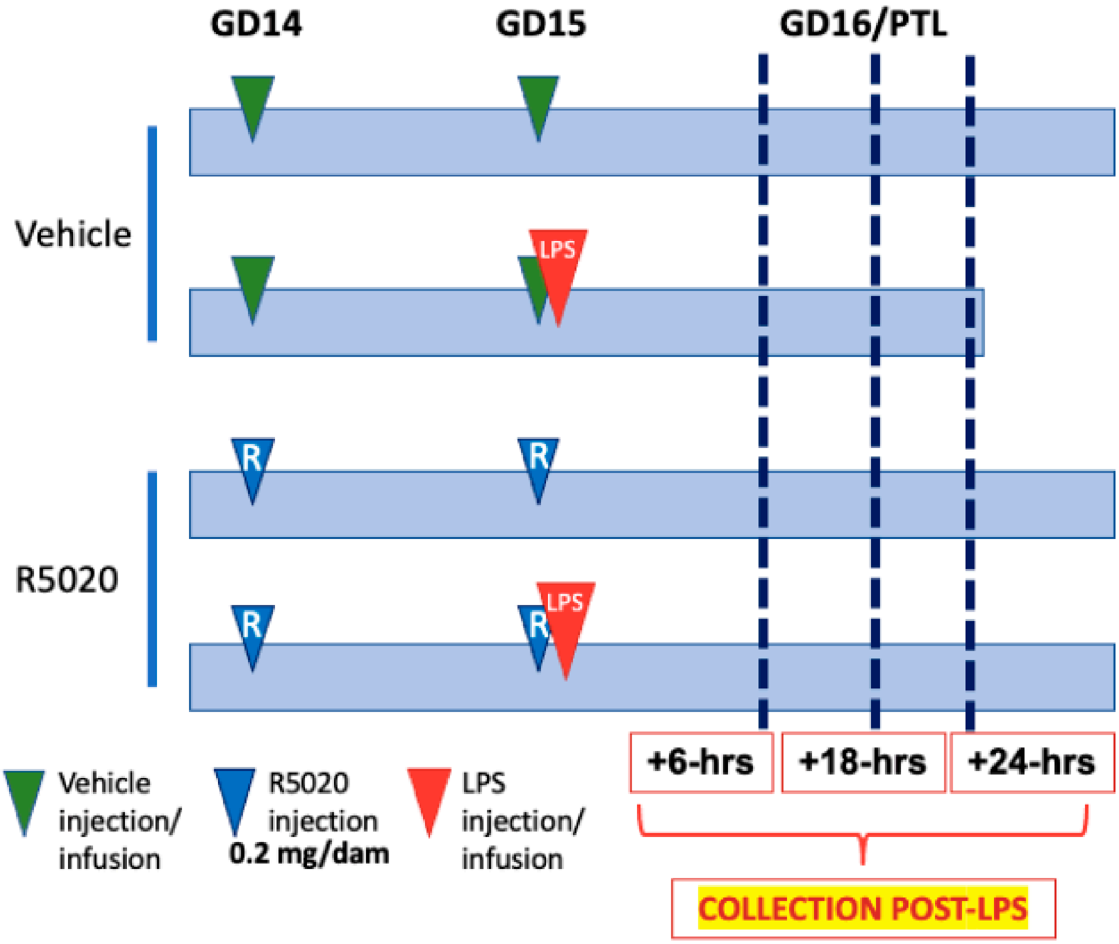

Figure S2. The scheme of drugs administration and sample collections in PTL mouse models. Schematic of gestational time points when Vehicle or R5020 and LPS were administered to pregnant mice and when tissues were collected. Dotted lines represent timepoints of cervical tissue collection. Gestational Days at which Vehicle was administered are marked by green triangles, R5020 administration – by blue triangles, and LPS injections – by red triangles.

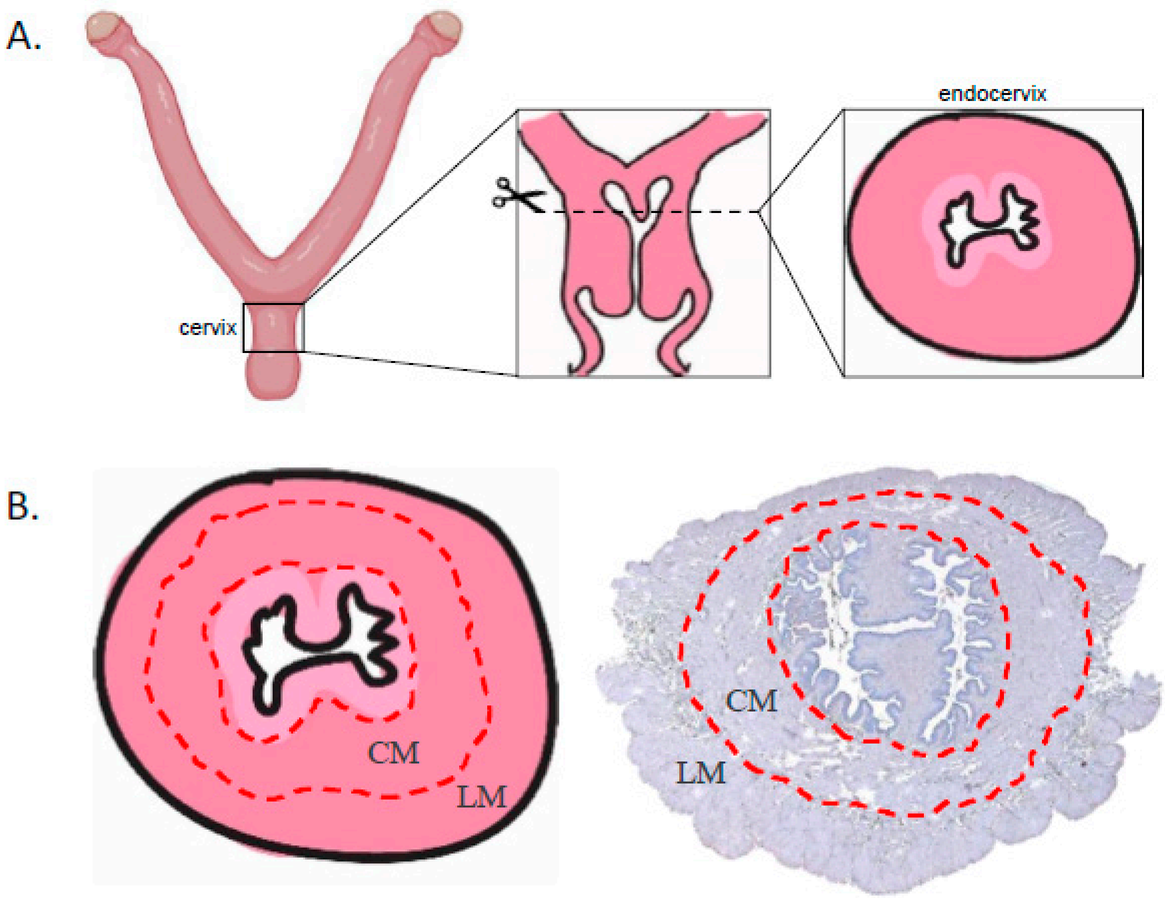

Figure S3. Smooth muscle layers in the transverse section of the murine endocervix. (**A**) mouse endocervix region. (**B**) Endocervical layer was divided into a longitudinal muscle layer (LM) and a circular muscle layer (CM).

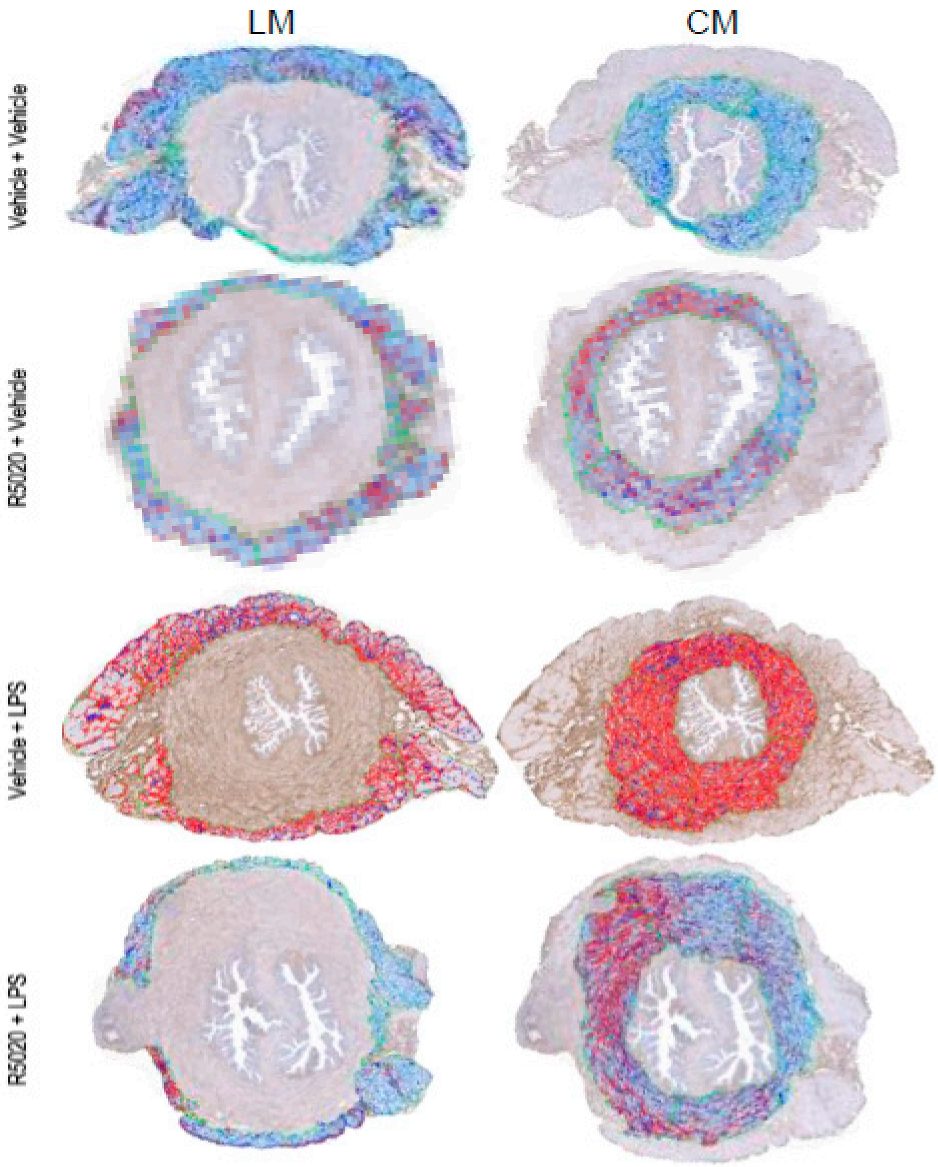

Figure S4. Representative images of longitudinal and circular smooth muscle layers of murine endocervix analyzed by Visiopharm software. Shown are images of HABP protein immunostaining in the local uterine inflammation model (magnification is 12.5x). Red area represents positive brown staining. Blue area represents nuclei.

Table S1: Primer pair information for mouse genes examined using qRT-PCR.

	SYMBOL	GENBANK ASCENSION (NM)	PRIMERS (5′-3′)	
			FORWARD	REVERSE
Reference genes	***Gapdh***	08084.3	AGGAGTAAGAAACCCTGGACCA	AGGCCCCTCCTGTTATTATGG
	Hprt	013556.2	CAGTCCCAGCGTCGTGAT	CAAGTCTTTCAGTCCTGTCCATAA
	Ppia	008907.1	CACCGTGTTCTTCGACATCA	CCAGTGCTCAGAGCTCGAAAG
	Tbp	013684	TCCCAAGCGATTTGCTGCAGTCATC	ACTCTTGGCTCCTGTGCACACCA
P4-metabolizing enzymes	*Akr1c18*	134066	TTTGGCACCTATGCAACTGAA	CAACTCTGGACGATGGGAAGT
	Srd5a1	175283	GAGTTGGATGAGTTGCGCCTA	GGACCACTGCGAGGAGTAG
Markers of cervical ripening	*Col1a1*	007742	GCTCCTCTTAGGGGCCACT	CCACGTCTCACCATTGGGG
	Col3a1	009930	CTGTAACATGGAAACTGGGGAAA	CCATAGCTGAACTGAAAACCACC
	Has2	008216	TGTGAGAGGTTTCTATGTGTCCT	ACCGTACAGTCCAAATGAGAAGT
Markers of Inflammation	*Ccl2*	011333	AGGTGTCCCAAAGAAGCTGTA	TCTGGACCCATTCCTTCTTG
	Cxcl1	008176	CCTGCAGACCATGGCTGGGAT	GTGTGGCTATGACTTCGGTTTGGG
	Il1b	008361	GGACCCCAAAAGATGAAGGGCTGC	GCTCTTGTTGATGTGCTGCTGCG
	Il6	031168	CCTCTCTGCAAGAGACTTCC	GCTCTTGTTGATGTGCTGCTGCG
